# Biological Evaluation and Molecular Docking Studies of Novel 1,3,4-Oxadiazole Derivatives of 4,6-Dimethyl-2-sulfanylpyridine-3-carboxamide

**DOI:** 10.3390/ijms23010549

**Published:** 2022-01-04

**Authors:** Piotr Świątek, Teresa Glomb, Agnieszka Dobosz, Tomasz Gębarowski, Kamil Wojtkowiak, Aneta Jezierska, Jarosław J. Panek, Małgorzata Świątek, Małgorzata Strzelecka

**Affiliations:** 1 Department of Medicinal Chemistry, Faculty of Pharmacy, Wroclaw Medical University, Borowska 211, 50-556 Wroclaw, Poland; malgorzata.strzelecka@umw.edu.pl; 2 Department of Medical Science Foundation, Faculty of Pharmacy, Wroclaw Medical University, Borowska 211, 50-556 Wroclaw, Poland; agnieszka.dobosz@umw.edu.pl; 3 Department of Biostructure and Animal Physiology, Wroclaw University of Environmental and Life Sciences, Kożuchowska 1/3, 51-631 Wroclaw, Poland; tomasz.gebarowski@upwr.edu.pl; 4 Faculty of Chemistry, University of Wroclaw, F. Joliot-Curie 14, 50-383 Wrocław, Poland; kamil.wojtkowiak@chem.uni.wroc.pl (K.W.); aneta.jezierska@chem.uni.wroc.pl (A.J.); jaroslaw.panek@chem.uni.wroc.pl (J.J.P.); 5 Hospital Pharmacy, University Clinical Hospital, Borowska 213, 50-556 Wrocław, Poland; mswiatek@usk.wroc.pl

**Keywords:** dimethylpyridine, 1,3,4-oxadiazole, cyclooxygenase, cytotoxicity, molecular docking

## Abstract

To date, chronic inflammation is involved in most main human pathologies such as cancer, and autoimmune, cardiovascular or neurodegenerative disorders. Studies suggest that different prostanoids, especially prostaglandin E_2_, and their own synthase (cyclooxygenase enzyme-COX) can promote tumor growth by activating signaling pathways which control cell proliferation, migration, apoptosis, and angiogenesis. Non-steroidal anti-inflammatory drugs (NSAIDs) are used, alongside corticosteroids, to treat inflammatory symptoms particularly in all chronic diseases. However, their toxicity from COX inhibition and the suppression of physiologically important prostaglandins limits their use. Therefore, in continuation of our efforts in the development of potent, safe, non-toxic chemopreventive compounds, we report herein the design, synthesis, biological evaluation of new series of Schiff base-type hybrid compounds containing differently substituted *N*-acyl hydrazone moieties, 1,3,4-oxadiazole ring, and 4,6-dimethylpyridine core. The anti-COX-1/COX-2, antioxidant and anticancer activities were studied. Schiff base **13**, containing 2-bromobenzylidene residue inhibited the activity of both isoenzymes, COX-1 and COX-2 at a lower concentration than standard drugs, and its COX-2/COX-1 selectivity ratio was similar to meloxicam. Furthermore, the results of cytotoxicity assay indicated that all of the tested compounds exhibited potent anti-cancer activity against A549, MCF-7, LoVo, and LoVo/Dx cell lines, compared with piroxicam and meloxicam. Moreover, our experimental study was supported by density functional theory (DFT) and molecular docking to describe the binding mode of new structures to cyclooxygenase.

## 1. Introduction

In recent years, the hybridization strategy has gained a noticeable attention in developing new medications. Hybrid molecules are designed through fusing at least two active pharmacophores in a single-hybrid molecule to improve the biological efficacy and minimize the possible toxicity relative to the parent drug. Hybridization strategy has been widely used to develop new anti-inflammatory drugs [1,2,3].

Inflammation is defined as a complex dynamic defensive process, in which the body responds to injury, infection via microbes, trauma, or toxins in the vascularized tissues. The influx of various cells of the host immune system (e.g., leukocytes, macrophages, mast cells), and release of numerous substances called inflammatory mediators (such as cytokines, free radicals, and eicosanoids, mainly prostaglandins) to the site of damage lead to pronounced vascular changes, including vasodilation, increased permeability and the formation of local edema, and the pain reaction associated with irritation of the pain receptors. This sequential cascade of events, both vascular and cellular, is responsible for the processes of repair, healing and reconstituting of damaged tissue. In pathological conditions the impairment of the effectiveness of repair mechanisms may be a major factor in the progression of various chronic diseases and disorders, including asthma, diabetes, cancer, cardiovascular diseases, arthritis, and inflammatory bowel disease [4,5,6].

Non-steroidal anti-inflammatory drugs (NSAIDs) are a diverse class of medicines commonly used for the treatment of acute and chronic inflammatory conditions, pain, and fever. Both their benefits and side effects arise due to inhibition of cyclooxygenase enzyme (COX) that catalyzes the conversion of arachidonic acid into prostaglandin H_2_ (PGH_2_), a precursor for the synthesis of biologically active prostanoids (prostaglandins PGD_2_, PGE_2_, PGF_2a_, prostacyclin PGI_2_, and thromboxane TxA_2_). Currently, it is documented that there are, at least two COX isoforms, COX-1 and COX-2. The role of COX-1 (constitutively expressed in most tissue types) and COX-2 (generally considered as inducible) is complex and depends on many factors, mainly the organ in which it acts. It is well-known that therapy with non-selective COX inhibitors is associated with a number of side effects including gastrointestinal erosions, renal failure, the exacerbation of hypertension, sodium and water retention. That toxicity is attributed to the inhibition of the COX-1-mediated generation of the cytoprotective prostanoids, such as PGE_2_ and PGI_2_. The dissimilarity in the structure of COX-1 and COX-2 isoenzyme resulted in the development of selective COX-2 inhibitors referred to as coxibs. COX-2 expression is greatly increased at inflammatory sites in response to cytokines such as interferon, TNFα, IL1, hormones, growth factors and hypoxia. The introduction of COXIBs offered an efficacious alternative to non-selective NSAIDs with improved gastrointestinal tolerability. However, long-term placebo-controlled studies revealed cardiovascular side effects that led to the withdrawal of some COXIBs from the American and European markets [7,8,9]. Due to the complexity of inflammation, blocking the synthesis of prostanoids is not able to completely limit this process, therefore new compounds are also tested for multidirectional effects on other components, including the generation of reactive oxygen and nitrogen species, modulation and neutralization of inflammatory cytokines and biogenic amines released by stimulated inflammatory cells [5,10]. This pleiotropic approach could be the key to more effective inhibition of inflammation.

Over the past decades, it has been suggested that NSAIDs may have possible anti-cancer activity. This effect is attributed to the inhibition of COX enzymes, especially COX-2 activity, the overexpression of which in various neoplastic tissues promotes proliferation and inhibits apoptotic death of cancer cells, stimulates angiogenesis, and increases the ability to invade the tumor. These observations have led the researchers to study specific COX-2 inhibitors as chemopreventive and potential chemotherapeutic agents [4,11,12,13].

Compounds with a hydrazide-hydrazone pharmacophore moiety, otherwise known as *N*-acyl hydrazones, constitute a class of organic compounds that have great potential in medicinal chemistry in the design of new drugs. They contain an azomethine group (–NH–N=CH–) bound to the carbonyl group that is responsible for their multidirectional biological applications. In addition, this group can play a significant role as an organic intermediate for the synthesis of new, more complex compounds, e.g., heterocyclic. The versatility of *N*-acyl hydrazones in medical chemistry is based on the ease of their synthesis, as they are usually formed in a condensation reaction between aldehydes or ketones with hydrazides [14,15,16]. Literature studies show that the *N*-acyl hydrazone moiety is characterized by various activities, e.g., anticancer [17,18,19,20], antimicrobial [21,22,23,24], anticonvulsant [25]. The analgesic and anti-inflammatory effect seems to be particularly important [26,27], and researchers have shown that many of the derivatives exhibit a mechanism of action related to the inhibition of cyclooxygenase [28,29]. This may result, firstly, from the relative acidity of the amide hydrogen of *N*-acyl hydrazone group, and secondly, from the ability of this group to stabilize free radicals. In addition, *N*-acyl hydrazone moiety has a structural similarity to *bis*-allylic moiety of unsaturated fatty acids, e.g., arachidonic acid [30,31,32].

The 1,3,4-oxadiazole ring, due to its properties, is an important element in the development of new drugs [33]. It is a bioisostere for carbonyl-containing derivatives, and is also a valid pharmacophore component, increasing the ability of oxadiazole-containing molecules to bind to a various ligands (e.g., muscarinic receptors, benzodiazepine receptors, dopamine, serotonin and norepinephrine transporters) [34,35,36,37,38,39,40,41]. According to the literature, compounds containing 1,3,4-oxadiazole moiety have confirmed various biological effects, including anticancer [42,43], antimicrobial [44,45,46,47], antidepressant [48] and anticonvulsant [49]. Particularly noteworthy is their analgesic and anti-inflammatory activity [50,51], in many cases associated with the inhibition of cyclooxygenase [52,53,54].

**Figure 1:**
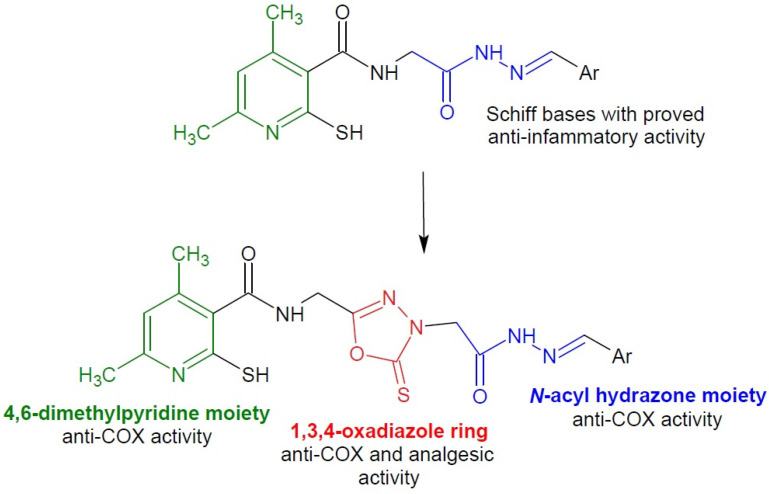
Scheme of the hybridization concept with the activity of individual structures.

In our previous paper, we have reported the synthesis of twelve different Schiff base derivatives of *N*-(2-hydrazinyl-2-oxoethyl)-4,6-dimethyl-2-sulfanylpyridine-3-carboxamide (Figure 1) [55]. The compounds were tested for anti-COX, antioxidant and anticancer activity. The derivatives showed promising properties, therefore we decided to obtain analogous structures additionally containing the pharmacophoric ring of 1,3,4-oxadiazole. The heterocyclic moiety may enhance the pharmacological action of the entire molecule.

Based on the above information, it can be concluded that our newly synthesized derivatives, which are hybrid compounds connecting the structure of 4,6-dimethylpyridine, 1,3,4-oxadiazole ring and *N*-acyl hydrazone moiety, may exhibit significant anti-inflammatory and chemopreventive effects, and their mechanism of action may be associated with anti-COX, antioxidant and anticancer activities (Figure 1). Considering that the binding pocket of COX-2 isoenzyme is bigger than that of COX-1, we expect that the extensive structure of new molecules will enhance the COX-2 selectivity compared to the previous Schiff bases, which inhibited COX-1 at lower concentrations than COX-2 [56].

The new compounds were examined as prospective COX-1/COX-2 inhibitors. Moreover, antioxidant and cytotoxic properties against A549 (pulmonary basial cell alveolar adenocarcinoma), MCF-7 (breast adenocarcinoma), LoVo (colon adenocarcinoma), and its drug resistant subline LoVo/Dx cell lines were studied to check the chemopreventive potential of the compounds. In addition, the structure study was performed on the basis of density functional theory (DFT) to investigate the energetic properties of the studied compounds depending on their conformation. Finally, molecular docking was carried out in order to suggest the binding mode and correlate it with biological activity.

## 2. Results and Discussion

### 2.1. Chemistry

**Scheme 1:**
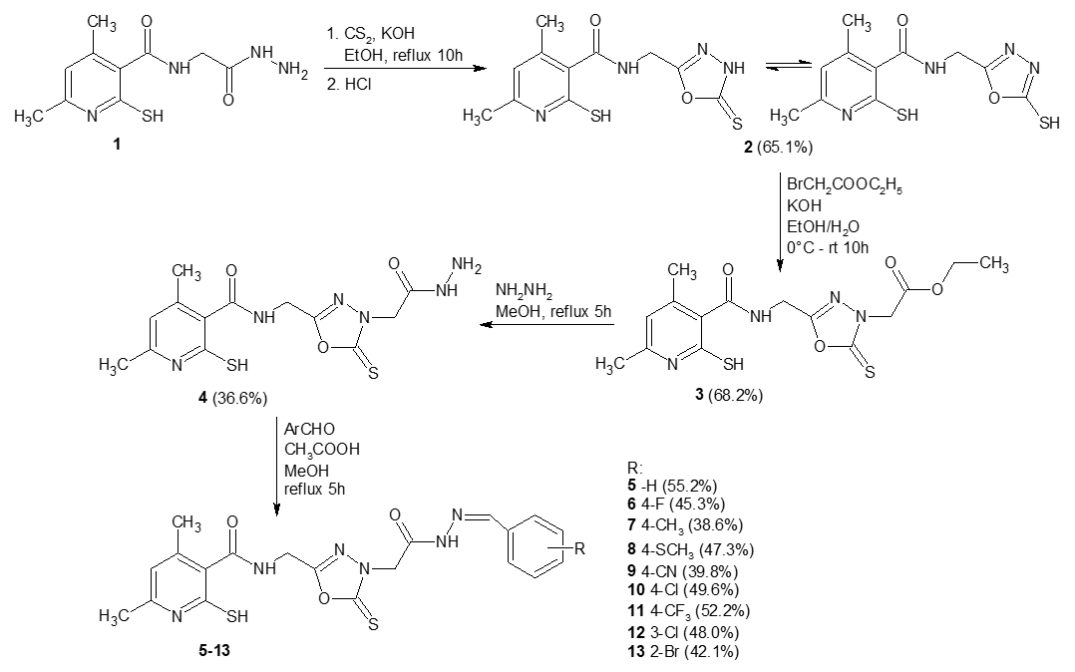
Scheme of the synthesis of new compounds **5**–**13** with the yields in brackets.

The synthesis of *N*-(2-hydrazinyl-2-oxoethyl)-4,6-dimethyl-2-sulfanylpyridine-3-carboxamide **1** was performed according to the protocols published previously [19]. Scheme 1 presents the synthesis of compounds which have not been described in the literature yet. Analytical and spectroscopic properties of all newly obtained derivatives were in good agreement with their predicted structures and are summarized in the experimental section. Initially, compound **1** was heated at reflux in the presence of carbon disulfide in basic conditions in ethanol. Subsequently, the reaction mixture was poured onto crushed ice and acidified with hydrochloric acid. As a result, hydrazide **1** was subjected to intramolecular cyclization and formation of a five-membered 1,3,4-oxadiazol-2-thione ring. In the next step, compound **2** was reacted with ethyl bromoacetate in a water-ethanolic potassium hydroxide solution. Thus, the ester derivative **3** was obtained. Subsequently, compound **3** was converted into hydrazide **4** by reaction with hydrazine hydrate. The synthesis of nine different Schiff base derivatives of compound **4** was carried out by the reaction of compound **4** with several aromatic aldehydes in methanol in the presence of a catalytic amount of acetic acid with good yields. The purity of the synthesized compounds was checked by elemental analyses. The structures of the various synthesized compounds were determined based on spectral data analysis, such as FT-IR and ^1^H NMR and ^13^C NMR.

The FT-IR spectrum of hydrazide **4** showed peaks at 1648 and 1623 cm^−1^ due to two carbonyl functions derived from the amide and hydrazide structure. Additionally, the IR spectra of compounds **4** and **5**–**13** exhibited in the 3294–3035 cm^−1^ range, the NH_2_ and NH weak band of the NHNH_2_, CONH and CONH-N= functions.

The ^1^H NMR spectrum of hydrazide **4** displayed no signals belonging to the OCH_2_CH_3_ group; instead, new signals derived from the hydrazide structure appeared at 6.07 (NHN**H_2_**) and 9.29 (N**H**NH_2_) ppm integrating for two protons and one proton, respectively. The ^1^H NMR spectra of compounds **5**–**13** displayed additional signals due to the azomethine group and the aromatic ring derived from the aldehyde moiety at the aromatic region, while the signal belonging to NH_2_ group of the hydrazide structure did not appear.

It should be noted that the ^1^H NMR spectra of compounds **5**–**13** show double signals corresponding to two amidic NH protons, azometine moiety protons and methylene protons. According to the literature, compounds having an arylidene-hydrazide structure may exist as *E*/*Z* geometrical isomers around the C=N double bond and as *cis*/*trans* amide conformers. It has been reported that when compounds containing an imine bond are dissolved in dimethyl-*d_6_* sulfoxide, they are present in the form of a geometrical *E* isomer. The *Z* isomer can be stabilized in less polar solvents by an intramolecular hydrogen bond [57,58,59,60]. In this study, the spectral data were obtained in dimethyl-*d_6_* sulfoxide solution and no signal belonging to *Z* isomer was observed. On the other hand, the *cis-trans* conformers of *E* isomer were present in the dimethyl-*d_6_* sulfoxide solution of compounds **5**–**13**. In the ^1^H NMR spectra of **5**–**13**, in particular, two sets of signals each belonging to the CH_2_ group, N=CH group and CONH group of *cis* and *trans* conformers were observed between 3.96 and 4.44, 7.96 and 8.54, 11.56 and 11.99 ppm respectively. The upfield lines of protons of the listed groups were assigned to the *cis*-conformer of the amide structure, while the downfield lines of the protons of the same groups were assigned to the *trans*-conformer of the amide structure [59].

### 2.2. Biological Tests 

#### 2.2.1. Cyclooxygenase Inhibition Assay

The compounds were studied for their potencies to inhibit COX-1 and COX-2 enzymes by the colorimetric inhibitor screening assay. The IC_50_ values (i.e., the concentration of tested compounds (μM) that can exert 50% inhibition of the enzyme activity) and the COX-2/COX-1 selectivity ratios were calculated after 2 min of incubation for each investigated and reference compound (piroxicam and meloxicam).

As can be seen from the data presented in Table 1, hydrazide **4** and its 4-methylsulfanylbenzylidene derivative **8** showed only COX-1 inhibitory activity, while the compound with 4-chlorobenzylidene moiety **10** selectively inhibited the activity of COX-2 isoenzyme. Four of the Schiff bases **5**, **11**, **12** and **13** with unsubstituted benzylidene moiety or containing 4-trifluoro, 3-chloro, 2-bromo groups, respectively exhibited higher anti-COX-1 activity compared with piroxicam and meloxicam. What is more, compound **13** inhibited the activity of COX-2 at a lower concentration than standards, and its COX-2/COX-1 selectivity ratio was similar to meloxicam.

#### 2.2.2. MTT Assay

The MTT (3-[4,5-dimethylthiazol-2-yl]-2,5-diphenyl tetrazolium bromide) assay was used to determine the cytotoxicity of tested compounds towards different cell lines: normal human dermal fibroblasts (NHDF), kidney epithelial cells (VERO), fibroblasts from Chinese hamster lung (V79) and four human cancer cells: A549 (pulmonary basal cell alveolar adenocarcinoma), MCF-7 (breast adenocarcinoma), LoVo (colon adenocarcinoma), and its drug resistant subline LoVo/Dx. The cell lines used in this study express COX-1 and COX-2. The LoVo line expressed COX-1, the LoVo/Dx subline express both COX-1 and COX-2 [61]. The A549 and MCF-7 lines shows expression of both enzymes [62,63]. 

This assay is suitable for the measurement of drug sensitivity in cultured cells, specified as the concentration of the compound required to achieve 50% growth inhibition, compared to the growth of the control cultured without any drug (50% inhibitory concentration, IC_50_) [64]. The results of the experiment were made for screening purpose, and are presented in Table 2.

Firstly, the effect of the new compounds on healthy cells (NHDF, V79 and VERO) were performed. With regards to NHDF cells, both, hydrazide **4** and all of the Schiff bases exhibited similar toxicity (IC_50_ in the range of 57.47–59.9 μM). The VERO line exhibited more differences in cells sensitivity: there was no toxicity caused to VERO cells by standard compounds (piroxicam and meloxicam) and IC_50_ for tested compounds was in the range 50.38 μM for compound **8** to 129.8 μM for compound 10. The results for V79 cell line were more similar to NHDF cells: IC_50_ was in the range 47.70 μM (for compound **7**) to 84.30 μM (for compound **5**).

The anti-cancer activity of the tested compounds was established towards A549 cell line. A549 cells are adenocarcinomic human alveolar basal epithelial cells, used as a model of lung adenocarcinoma [65]. Inhibitory concentrations caused 50% of growth inhibition for these cells were similar for all tested compounds (3.16–4.34 μM), and was 25 to 35 times lower than that of piroxicam and meloxicam. The next tested cell line was MCF-7 from human breast adenocarcinoma. In this case, the standard compounds possessed no antitumor activity in the range of used concentrations, while the inhibitory concentrations of tested compounds were in the range of 6.33–9.63 μM. Antitumor activity towards LoVo cells (from human colon) was similar as for A549 cells, and the inhibitory concentrations were 34- to 45-fold lower than that of piroxicam and meloxicam. The drug resistant subline LoVo/Dx exhibited more different sensitivity to the tested compounds. Hydrazide **4** and five Schiff bases **5**, **6**, **7**, **9** and **13** with unsubstituted benzylidene moiety or containing 4-fluoro, 4-methyl, 4-cyano and 2-bromo groups, respectively inhibited the growth of LoVo/Dx cells in the concentrations 13- to 22-fold lower than the standards.

The performed investigation revealed that all tested compounds had more significant anticancer activity than piroxicam and meloxicam. Moreover, the therapeutic index, i.e., the ratio of the concentrations that inhibit 50% of healthy and cancerous cells (A549, MCF-7 and LoVo cell lines) was high for all investigated compound, and the IC_50_ values for tumor cells were 1.3- and even 27-fold lower than for normal cells (Table 3). 

#### 2.2.3. Evaluation of Reactive Oxygen Species (ROS) Level Inside the Cells

ROS participates in various redox-regulatory mechanisms of cells to maintain homeostasis. When the system’s ability to neutralize and eliminate free radicals and active intermediates fails, the intracellular redox potential shifts towards oxidative stress leading to DNA mutations, which can further promote neoplastic transformation. A chronic inflammation is the main system capable of inducing an oxidative stress. Prostaglandins induce the expression of certain inflammatory cytokines, which can, in turn, enhance the production of ROS. The inhibition of these paths at an early stage of neoplasia may be a critical moment in the process of chemoprevention. [66]. Taking this into account, the next part of the investigations was the ROS scavenging activity of studied compounds examined under or without the influence of oxidative stress, generated by the presence of H_2_O_2_ inside the cell cultures. As in the previous tests, two oxicams (meloxicam and piroxicam) were taken as the reference drugs (Table 4 and Table 5) due to their proven ability to scavenging ROS [67,68,69]. 

Two cell lines were chosen for investigations: V79 (Chinese hamster lung fibroblasts) and LoVo. V79 normal cells are widely used as the cell line tested in the studies of the oxidative stress [70,71,72]. These cells are a living system model to explain the mechanism of ROS effects caused by varied compounds [73].

The tests established on V79 cell line revealed that among ten tested compounds, two of them (hydrazide **4** and Schiff base **13** containing 2-bromobenzylidene group) showed significant ROS scavenging activity under normal conditions almost two times higher than the standard drugs. The same compounds exhibited the best values during oxidative stress, induced by H_2_O_2_ (Table 4). 

**Figure 2:**
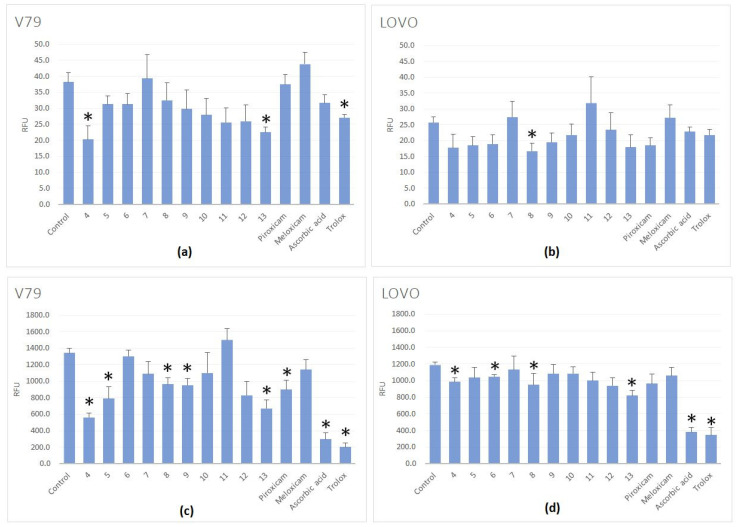
ROS levels in V79 and LOVO cells incubated in the presence of tested compounds (in concentration of 100 μM): untreated with H_2_O_2_ (**a**,**b**) and treated with H_2_O_2_ (100 μM, 30 min) (**c**,**d**). Results are presented as RFU (relative fluorescent units) of fluorescence levels. The statistical significance of the differences between the results for the tested compounds, compared to the control, was calculated using Tukey’s post hoc test (* *p* < 0.05).

Considering the test on LoVo line 4-methylsulphanylbenzylidene derivative (**8**) showed high ROS scavenging activity under normal conditions. On the other hand the best activity during oxidative stress characterized compounds **4** and **13** as in the case of the first cell line V79 (Table 5, Figure 2).

Comparing the antioxidant activity of our ten tested compounds to standard ROS scavengers: trolox and ascorbic acid, we can see that the activity of all compounds tested on V79 line, is lower than trolox and ascorbic acid (Table 4). Trolox and ascorbic acid antioxidant activities were previously tested by Szczęśniak-Sięga et al. on V79 cell line in the similar conditions, and the values of their mean E/E_0_ were similar to our results [74]. The same situation is observed for LoVo line, where the antioxidant activity of tested compounds were also lower than for standard scavengers, but very similar for all tested compounds (Table 5).

According to the results, it can be seen that our tested compounds can protect healthy cells against oxidative stress to a greater extent than cancer cells.

### 2.3. Static Density Functional Theory (DFT) Models and Molecular Docking Study 

Molecular modeling techniques are useful in development of the interaction models between ligands and receptors. Within this study we applied diverse methods to characterize the receptors (sequence similarity evaluation), the ligands themselves (density functional theory, DFT) and their interactions with protein receptors (docking studies). We will start with the discussion of the protein models. For human COX-1 (PDB-Protein Data Bank code: 6Y3C [75]), the sequence similarity to ovine protein model (PDB code: 4O1Z [76]) was equal to 91% and for the human COX-2 (PDB code: 5KIR [77]) the resemblance to the murine model (PDB code: 4M11 [76]) reached 88%—the sequence alignments are presented in Figure S1 of Supplementary Material. Sequence homology between human receptors was close to 66%, hence the different mode of binding, as well as affinity energies, were anticipated. Significant homology in the binding places between COX-1 orthologes was seen—the binding site of the protein structure with 6Y3C code (apo form, with no ligand molecule) was inferred from knowledge of the 4O1Z binding pocket-molecular docking has shown that our assumption was correct and even if affinity energies for human COX-1 were slightly lower, the docking procedure itself was successful. Residues such as Leu352, PhE-518, Ser530, Trp387 and Val349 were involved in the direct interactions with ligands in all the studied receptors.

**Figure 3:**
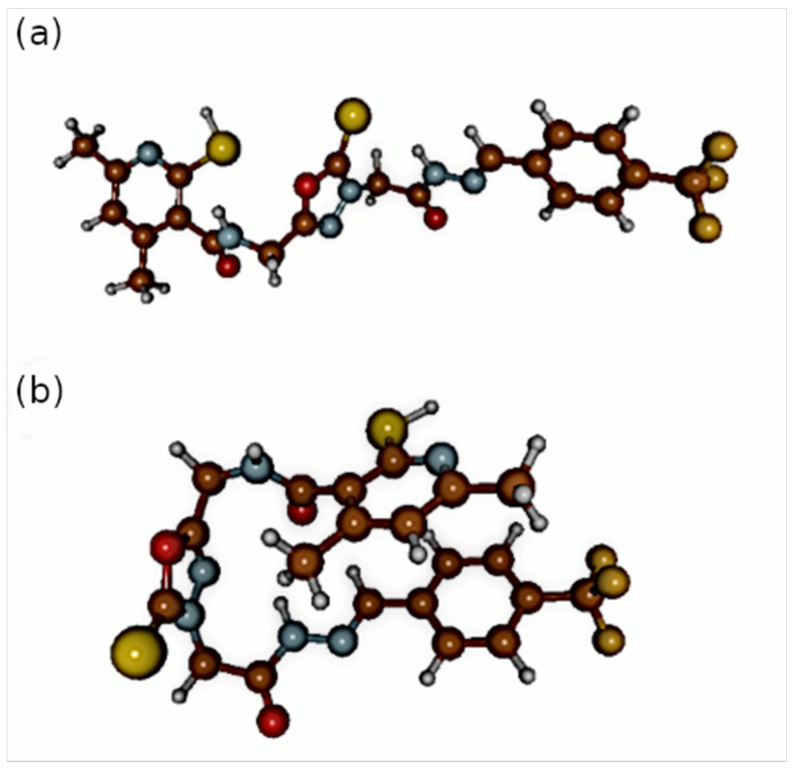
Optimized structures of the compound denoted by number **11**. The atoms coloring scheme is as follows: brown-carbon, blue-nitrogen, red-oxygen, yellow-sulphur, orange-fluorine and white-hydrogen; (**a**) extended conformation of **11** (**b**) sandwich-like conformation of **11**.

Two types of conformations of the studied set of compounds were found in the DFT structural optimizations: one that assumed more packed, sandwich-like structure—denoted by additional “S” in brackets after the compound number—and the second one which was more extended (with “E” in brackets after the compound number). The details are shown in the Figure 3 and in the Supplementary Materials (Table S1). 

The structures exhibited diverse features, including intramolecular N-H…O hydrogen bonds and non-covalent stacking-like interactions between the phenyl and pyridine rings, which affected the located conformations and their stability. The set of compounds shares the common part and differs generally only by the phenyl ring substitution. The applied energetic criterion—relative energy between the conformers of a given compound—shows that the sandwich-like structures are in general ca. 8–10 kcal/mol lower in energy. This energy separation between conformers was found in the gas phase DFT study based on different initial arrangement of the central 1,3,4-oxadiazole ring. Both types of conformations were then examined via the docking approach.

**Figure 4:**
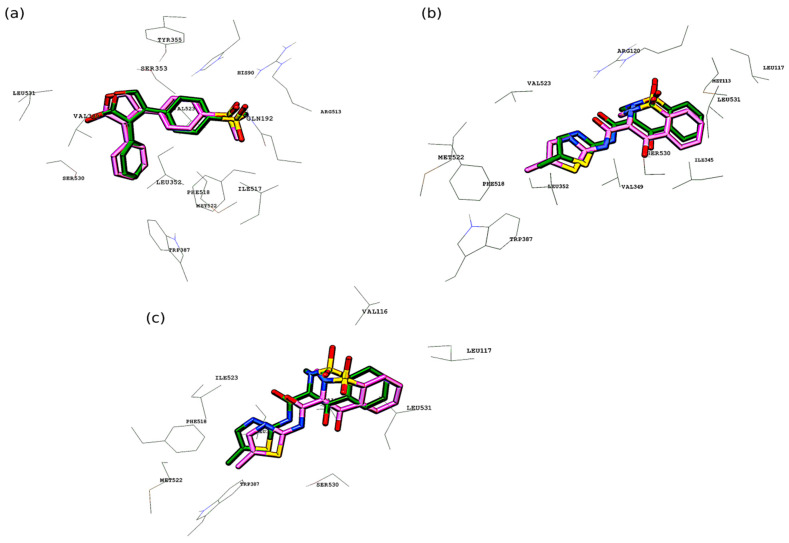
Validation of the docking protocol. Alignment of co-crystallized (pink carbon atoms) and docked structures (green carbon atoms) of: (**a**) RCX to COX-2 (5KIR), (**b**) MXM to COX-2 (4M11), (**c**) MXM to COX-1 (4O1Z).

In the next step, the protein-ligand complex was analyzed using the flexible docking method. The idea of the flexible docking is that both the ligand and the selected receptor residues are treated as conformationally flexible. The initial conformation of the ligand is modified to adapt to the binding site and the selected residues of the binding site are also allowed to reorient. Initial docking validation was performed, and it consisted of re-docking of the ligands that were previously co-crystallized with the corresponding receptors. It was meloxicam (MXM) for both ovine COX-1 (4O1Z) and murine COX-2 (4M11), and rofecoxib (RCX) for COX-2 (5KIR) from *Homo sapiens*. For human COX-1 (6Y3C) validation was not possible because of lack of the co-crystallized ligand alongside the protein in the PDB repository. The validation set of the docked structures was very similar to the crystallized cases (see Figure 4): the RMSD (root-mean-square deviation) factor for the ligand position was equal to 1.418 Å for rofecoxib in 5KIR, 0.930 Å for meloxicam in 4M11 and 1.037 Å for meloxicam in 4O1Z. The RMSD was calculated based on the structures with the highest conformational similarity to the co-crystallized ligands. For every structure of the MXM and RCX, the lowest RMSD value was obtained for conformations with the second lowest affinity energy. 

**Figure 5:**
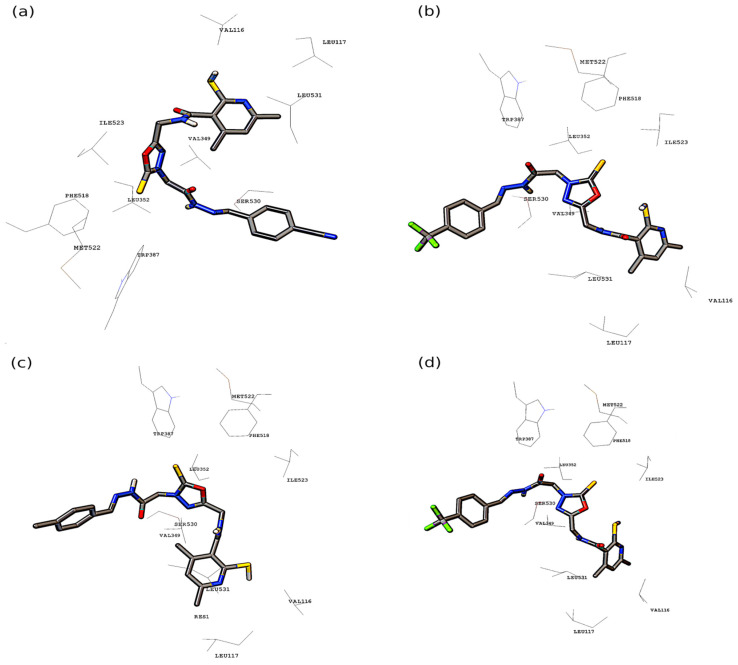
The most energetically favorable conformations of examined compounds in the active site of COX-1: (**a**) **9(S)** docked to ovine COX-1, (**b**) **11(E)** docked to human COX-1, (**c**) **7(E)** docked to ovine COX-1, (**d**) **11(S)** docked to human COX-1.

**Figure 6:**
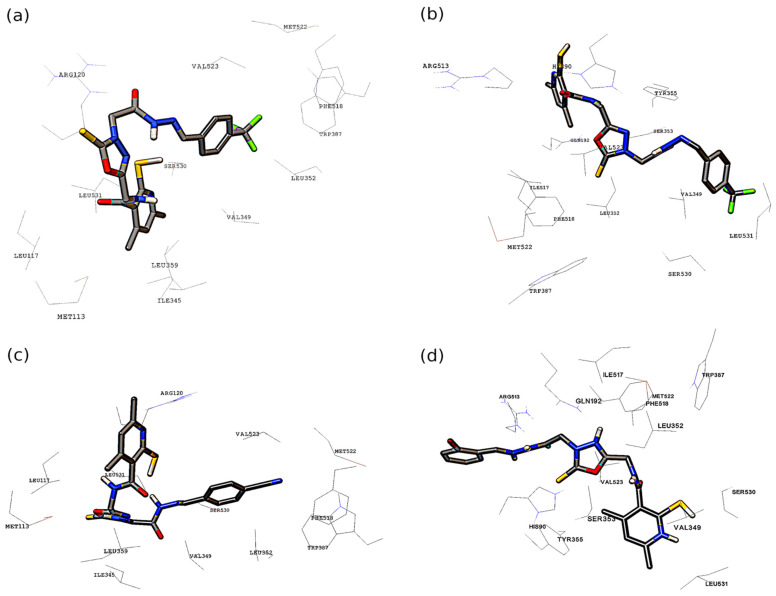
The most energetically favorable conformations of examined compounds in the active site of COX-2: (**a**) **11(S)** docked to murine COX-2, (**b**) **11(E)** docked to human COX-2, (**c**) **9(E)** docked to murine COX-2, (**d**) **13(S)** docked to human COX-2.

After successful validation of the docking protocol, the further characterization of the binding pocket interactions with the set of the studied molecules was performed. The complicated network of interactions, difficult to present in a 3D representation is provided in the 2D form in Figure S2 of the Supplementary Material. The most favorable structures with regards to the binding affinity energy (presented in Figure 5 and Figure 6) were chosen for detailed analysis. The most active extended-conformation compound towards the human COX-1 was **11(E)**. The stability of the binding of this compound by the human COX-1 can be explained by formation of hydrogen bonds between the Arg120, Ser530 and Asn375 residues and the ligand (depicted in Figure S2). Stabilizing non-covalent interaction (halogen bonding) was detected by the Discovery Studio Visualizer 2021 [78] between one of the fluorine substituents of the phenyl ring and the Phe-529 residue (also see Figure S2). Other non-covalent interactions present in the binding pocket are graphically presented in Figure S2. The structure represented by **11(S)** compound was also the most stabilized among the others of sandwich-like conformations. In this case, the most important modes of the binding contained also the hydrogen bonds between the ligand and the Arg120, Asn375 residues. Numerous other non-covalent interactions were detected, and they are presented in Figure S2.

It is worth noting that the docking to the apo structure of the human COX-1 was successful because of the use of the flexible docking protocol and previous characterization of the similarity of the binding sites with the use of bioinformatics tools (see Figure S1). For the ovine COX-1, the affinity energies were generally larger by ca. 2 kcal/mol for each compound comparing with the previous receptor (Table 6 and Table 7). The most selective extended structure was **7(E)** and the best among the sandwich-like structures was **9(S)**. For the **7(E)** compound a large number of π-π stacked, amide-π stacked, π-sulphur and π-σ interactions were present (see Figure S2). In the case of **9(S)** the π-alkyl, π-σ, alkyl and also π-sulphur interactions were the most significant. The binding energy for both **7(E)** and **9(S)** compounds exceeded -10.0 kcal/mol. The extended **11(E)** compound was found as the most active for the *Mus musculus* COX-2 (Table 8). The halogen bonds formation between fluorine substituents of ligand phenyl ring and Met522 residue was observed. The Arg120 residue and the nitrogen from the ligand pyridine ring were involved in the hydrogen bonding; non-covalent interactions are depicted in Figure S2. Its affinity towards *Mus musculus* COX-2 was even higher than that of the co-crystallized ligand-MXM. Among sandwich-like structures the most promising seems to be the **9(S)** molecule. In this case, the hydrogen bonds between the ligand and the residues Ser530 and Arg120 are present. Plethora of Van der Waals interactions between alkyl and aromatic parts of ligand and valine and leucine residues were also present (see Figure S2). The human COX-2 was bounded more efficiently by extended conformation of the examined compounds (Table 9). The differences were most significant for **11(E)** where the affinity energy between two investigated structures varied by more than 2 kcal/mol. The **11(E)** molecule in the binding pocket was stabilized by the network of intermolecular hydrogen bonds between the ligand and the Thr94, His90, Ser353, Tyr355 and Arg120 residues. Numerous non-covalent interactions were detected, and they are graphically presented in Figure S2. The **13(S)** molecule had the highest affinity towards the receptor among the sandwich-like structures. The major role was played by the amide-π stacked and π-σ as well as other non-covalent interactions present in the binding pocket (for details see Figure S2).

Concluding the docking study, we have found the substituent effect to change the affinity energy by 2–3 kcal/mol, which is a moderate but visible effect (ca. 20%). It was also found that the sandwich-like structures varied less in the affinity energies than the extended conformations. This is especially for the human COX-1, where only molecule conformations for structure **4** possessed lower affinity comparing to other compounds. The affinity energy differences between the most and the least favorable conformations of each of the ligands were ca. 3–4 kcal/mol. The affinities for the most stable structures varied even less. Generally speaking, the lower affinity energies were obtained when the docking procedure was applied to human and murine COX-2 receptors. The compound **4** was the least selective for the examined receptors, probably due to the lack of stabilizing non-covalent interactions provided by the phenyl ring and its substituents. It was most pronounced for receptors with 6Y3C, 4M11 and 5KIR PDB codes. The **11(S)** compound could be considered as potentially the most promising structure and it seems that the CF_3_ substituent of the phenyl ring plays an important role in stabilizing that ligand in the binding pocket (due to the intermolecular halogen bonds formation).

## 3. Materials and Methods

### 3.1. Chemistry

#### 3.1.1. Instruments and Chemicals

All solvents, reagents and chemicals used during experiments described in this paper were delivered by commercial suppliers (Alchem, Wrocław, Poland; Chemat, Gdańsk, Poland; Archem, Łany, Poland) and were used without further purification. Any dry solvents were received due to standard procedures. Reaction progress was monitored by the thin-layer chromatography (TLC) technique, on TLC plates made of 60-254 silica gel, and was visualised by UV light at 254/366 nm. Melting points of final compounds were determined on Electrothermal Mel-Temp 1101D apparatus (Cole-Parmer, Vernon Hills, IL, USA) using open capillary method, no correction needed. ^1^H NMR (300 MHz) and ^13^C NMR (75 MHz) spectra were recorded using Bruker 300 MHz NMR spectrometer (Bruker Analytische Messtechnik GmbH, Rheinstetten, Germany) in DMSO-*d_6_*, with tetramethylsilane (TMS) as an internal reference. Chemical shifts (δ) were reported in ppm. In order to record and read spectra, TopSpin 3.6.2. (Bruker Daltonik, GmbH, Bremen, Germany) program was used. Elemental analyses for carbon, nitrogen and hydrogen were carried out on a Carlo Erba NA 1500 analyzer and were within ±0.4% of the theoretical value. FT-IR spectra were measured on Nicolet iS50 FT-IR Spectrometer (Thermo Fisher Scientific, Waltham, MA, USA). Frequencies were reported in cm^−1^. All samples were solid, and spectra were read by OMNIC Spectra 2.0 (Thermo Fisher Scientific, Waltham, MA, USA).

#### 3.1.2. Preparation and Experimental Properties of Compounds **2**–**13**

The synthesis protocols and experimental data for compound **1** have already been reported [19].

Synthesis of 4,6-dimethyl-*N*-[(5-sulfanylidene-4,5-dihydro-1,3,4-oxadiazol-2-yl)methyl)-2-sulfanylpyridine-3-carboxamide **2**.

The hydrazide 1 (1.27 g, 0.005 mol) and KOH (0.56 g, 0.01 mol) were dissolved in ethanol (50 mL) in a round bottom flask. To this stirring mixture carbon disulphide (3 mL, 0.05 mol) was added and the whole was refluxed for 10 h till evolution of hydrogen sulfide was ceased. Then, the reaction mixture was cooled and slowly acidified with diluted hydrochloric acid. Formed precipitate was filtered off, washed with cold water, dried and recrystallized from ethanol giving white solid of compound **2**.

Yield: 65.1%, m.p.: 270–274 °C

FT-IR (selected lines, γ_max_, cm^−1^): 3155, 3028 (NH), 2888 (C-H aliph.), 1623 (C=O)

^1^H NMR (300 MHz, DMSO-*d_6_*): δ = 2.06 (s, 3H, CH_3_), 2.27 (s, 3H, CH_3_), 4.41–4.43 (d, 2H, CH_2_, *J* = 6 Hz), 6.49 (s, 1H, H-_pyridine_), 8.81 (t, 1H, NH, *J* = 6 Hz), 13.28 (s, 1H, SH), 14.40 (s, 1H, NH);

Synthesis of ethyl 2-(5-((2-sulfanyl-4,6-dimethylpyridine-3-carboxamido)methyl)-2-sulfanylidene-2,3-dihydro-1,3,4-oxadiazol-3-yl)acetate **3**.

A 100 mL round bottom flask containing 1.2 g (0.02 mol) of potassium hydroxide, 60 mL of ethanol, 6 mL of water was placed on a magnetic stirrer. Then, after dissolving the KOH, 2.96 g (0.01 mol) of compound **2** was added. The reaction was carried out at 0–5 °C. Then 1.1 mL (0.01 mol) of ethyl bromoacetate was added and left for 10 h on a magnetic stirrer. The resulting precipitate was filtered off and allowed to dry. The product **3** was then crystallized from ethanol.

Yield: 68.2%, m.p.: 155–160 °C.

FT-IR (selected lines, γ_max_, cm^−1^): 3185 (NH), 2984, 2930 (C-H aliph.), 1736 (C=O), 1640 (C=O).

^1^H NMR (300 MHz, DMSO-*d_6_*): δ = 1.15–1.21 (m, 3H, CH_3_), 2.05 (s, 3H, CH_3_), 2.26 (s, 3H, CH_3_), 4.11–4.13 (m, 2H, CH_2_), 4.18 (s, 2H, CH_2_), 4.54–4.56 (d, 2H, CH_2_, *J* = 6 Hz), 6.48 (s, 1H, H-_pyridine_), 8.82 (t, 1H, NH, *J* = 6 Hz), 13.28 (s, 1H, SH);

Synthesis of *N*-{[4-(2-hydrazinyl-2-oxoethyl)-5-sulfanylidene-4,5-dihydro-1,3,4-oxadiazol-2-yl] methyl}-4,6-dimethyl-2-sulfanylpyridine-3-carboxamide **4**.

In a 100 mL round bottom flask, 3.68 g (0.01 mol) of compound **3** and 30 mL of methanol were placed. The resulting mixture was refluxed with stirring. After the compound 3 had dissolved, 5.1 mL of hydrazine hydrate was added, and the mixture was heated for 5 h. The obtained precipitate was filtered off and allowed to dry. The compound **4** was then recrystallized in methanol.

Yield: 36.6%, m.p.: 201–205 °C.

FT-IR (selected lines, γ_max_, cm^−1^): 3294 (NH_2_), 3169, 3035 (NH), 1648 (C=O), 1623 (C=O)

^1^H NMR (300 MHz, DMSO-*d_6_*): δ = 2.06 (s, 3H, CH_3_), 2.27 (s, 3H, CH_3_), 3.80 (s, 2H, CH_2_), 4.53–4.55 (d, 2H, CH_2_, *J* = 6 Hz), 6.07 (s, 2H, NH_2_) 6.52 (s, 1H, H-_pyridine_), 8.70 (t, 1H, NH, *J* = 6 Hz), 9.29 (s, 1H, NH), 13.32 (s, 1H, SH);

General Procedure for Preparation of Compounds **5**–**13**.

An amount of 0.18 g (5 × 10^−4^ mol) of compound **4** and 25 mL of methanol were placed in a 100 mL round bottom flask. The obtained mixture was heated under reflux until the compound 4 has dissolved completely. Then 2 mL of acetic acid and 7.5 × 10^−4^ mol of appropriate benzaldehyde were added to the mixture. The mixture was heated for 5 h. The resulting precipitate was filtered off and allowed to dry, then crystallized from ethanol.

4,6-Dimethyl-*N*-{[4-(2-(2-benzylidenehydrazinyl)-2-oxoethyl)-5-sulfanylidene-4,5-dihydro-1,3,4-oxadiazol-2-yl]methyl}-2-sulfanylpyridine-3-carboxamide **5**.

Yield: 55.2%, m.p.: 236–239 °C.

FT-IR (selected lines, γ_max_, cm^−1^): 3245, 3043 (NH), 2928 (C-H aliph.), 1644 (C=O), 1626 (C=O).

^1^H NMR (300 MHz, DMSO-*d_6_*): δ = 2.06 (s, 3H, CH_3_), 2.27 (s, 3H, CH_3_), 4.00, 4.42 (2s, 2H, CH_2_), 4.53–4.55 (d, 2H, CH_2_, *J* = 6 Hz), 6.52 (s, 1H, H-_pyridine_), 7.41–7.43 (m, 3H, ArH), 7.67–7.68 (m, 2H, ArH), 8.01, 8.18 (2s, 1H, CH), 8.69 (t, 1H, NH, *J* = 6 Hz), 11.62, 11.71 (2s, 1H, NH), 13.36 (s, 1H, SH);

^13^C NMR (75 MHz, DMSO-*d_6_*): δ = 18.78, 19.76, 33.17, 33.87, 115.86, 127.57, 129.52, 130.74, 134.64, 137.08, 144.15, 146.84, 148.79, 152.45, 154.15, 164.15, 167.08, 169.52, 173.67, 188.55.

4,6-Dimethyl-*N*-{[4-(2-(2-(4-fluorobenzylidene)hydrazinyl)-2-oxoethyl)-5-sulfanylidene-4,5-dihydro-1,3,4-oxadiazole-2-yl]methyl}-2-sulfanylpyridine-3-carboxamide **6**.

Yield: 45.3%, m.p.: 295–298 °C.

FT-IR (selected lines, γ_max_, cm^−1^): 3206, 3056 (NH), 2932 (C-H aliph.), 1657 (C=O), 1627 (C=O).

^1^H NMR (300 MHz, DMSO-*d_6_*): δ = 2.06 (s, 3H, CH_3_), 2.27 (s, 3H, CH_3_), 3.99, 4.41 (2s, 2H, CH_2_), 4.53–4.55 (d, 2H, CH_2_, *J* = 6 Hz), 6.52 (s, 1H, H-_pyridine_), 7.24–7.30 (m, 2H, ArH), 7.72–7.76 (m, 2H, ArH), 8.00, 8.18 (2 s, 1H, CH), 8.69 (t, 1H, NH, *J* = 6 Hz), 11.63, 11.72 (2 s, 1H, NH), 13.36 (s, 1H, SH);

^13^C NMR (75 MHz, DMSO-*d_6_*): δ = 18.76, 19.73, 33.27, 33.92, 115.76, 127.53, 129.56, 130.77, 134.54, 137.11, 144.18, 146.83, 148.81, 152.35, 154.17, 164.15, 167.02, 169.55, 173.67, 188.52.

4,6-Dimethyl-*N*-{[4-(2-(2-(4-methylbenzylidene)hydrazinyl)-2-oxoethyl)-5-sulfanylidene-4,5-dihydro-1,3,4-oxadiazole-2-yl]methyl}-2-sulfanylpyridine-3-carboxamide **7**.

Yield: 38.6%, m.p.: 215–220 °C.

FT-IR (selected lines, γ_max_, cm^−1^): 3210, 3064 (NH), 2929 (C-H aliph.), 1646 (C=O), 1621 (C=O).

^1^H NMR (300 MHz, DMSO-*d_6_*): δ = 2.06 (s, 3H, CH_3_), 2.27 (s, 3H, CH_3_), 2.32 (s, 3H, CH_3_), 3.98, 4.40 (2 s, 2H, CH_2_), 4.53–4.55 (d, 2H, CH_2_, *J* = 6 Hz), 6.47 (s, 1H, H-_pyridine_), 7.55–7.57 (m, 2H, ArH), 7.74–7.76 (m, 2H, ArH), 7.98, 8.14 (2 s, 1H, CH), 8.69 (t, 1H, NH, *J* = 6 Hz), 11.56, 11.67 (2 s, 1H, NH), 13.36 (s, 1H, SH);

^13^C NMR (75 MHz, DMSO-*d_6_*): δ = 18.78, 19.76, 21.46, 33.27, 33.92, 115.61, 127.57, 129.03, 130.25, 134.51, 137.09, 141.96, 146.83, 148.81, 152.35, 154.17, 161.96, 165.86, 169.52, 173.62, 188.50.

4,6-Dimethyl-*N*-{[4-(2-(2-(4-methysulfanylbenzylidene)hydrazinyl)-2-oxoethyl)-5-sulfanylidene-4,5-dihydro-1,3,4-oxadiazole-2-yl]methyl}-2-sulfanylpyridine-3-carboxamide **8**.

Yield: 47.3%, m.p.: 235–238 °C.

FT-IR (selected lines, γ_max_, cm^−1^): 3197 (NH), 2916 (C-H aliph.), 1642 (C=O), 1620 (C=O).

^1^H NMR (300 MHz, DMSO-*d_6_*): δ = 2.06 (s, 3H, CH_3_), 2.27 (s, 3H, CH_3_), 2.52 (s, 3H, CH_3_), 3.98, 4.40 (2 s, 2H, CH_2_), 4.53–4.55 (d, 2H, CH_2_, *J* = 6 Hz), 6.52 (s, 1H, H-_pyridine_), 7.59–7.62 (m, 2H, ArH), 7.74–7.77 (m, 2H, ArH), 7.96, 8.13 (2 s, 1H, CH), 8.69 (t, 1H, NH, *J* = 6 Hz), 11.58, 11.67 (2 s, 1H, NH), 13.59 (s, 1H, SH);

^13^C NMR (75 MHz, DMSO-*d_6_*): δ = 14.63, 18.78, 19.76, 33.31, 33.87, 126.10, 127.81, 129.27, 130.74, 134.51, 137.09, 143.18, 147.03, 148.72, 152.35, 154.34, 161.23, 165.86, 169.52, 173.62, 187.23.

4,6-Dimethyl-*N*-{[4-(2-(2-(4-cyanobenzylidene)hydrazinyl)-2-oxoethyl)-5-sulfanylidene-4,5-dihydro-1,3,4-oxadiazole-2-yl]methyl}-2-sulfanylpyridine-3-carboxamide **9**.

Yield: 39.8%, m.p.: 298–300 °C.

FT-IR (selected lines, γ_max_, cm^−1^): 3202 (NH), 2918 (C-H aliph.), 2224 (CN) 1667 (C=O), 1620 (C=O).

^1^H NMR (300 MHz, DMSO-*d_6_*): δ = 2.06 (s, 3H, CH_3_), 2.27 (s, 3H, CH_3_), 4.02, 4.35 (2 s, 2H, CH_2_), 4.53–4.55 (d, 2H, CH_2_, *J* = 6 Hz), 6.52 (s, 1H, H-_pyridine_), 7.87 (s, 4H, ArH), 8.05, 8.24 (2 s, 1H, CH), 8.69 (t, 1H, NH, *J* = 6 Hz), 11.85, 11.96 (2 s, 1H, NH), 13.36 (s, 1H, SH);

^13^C NMR (75 MHz, DMSO-*d_6_*): δ = 18.69, 19.54, 33.20, 33.49, 97.18, 112.56, 116.22, 125.93, 128.43, 129.27, 133.22, 137.09, 143.18, 147.03, 148.72, 152.35, 154.34, 161.23, 167.50, 169.52, 187.52.

4,6-Dimethyl-*N*-{[4-(2-(2-(4-chlorobenzylidene)hydrazinyl)-2-oxoethyl)-5-sulfanylidene-4,5-dihydro-1,3,4-oxadiazole-2-yl]methyl}-2-sulfanylpyridine-3-carboxamide **10**.

Yield: 49.6%, m.p.: 278–280 °C.

FT-IR (selected lines, γ_max_, cm^−1^): 3333, 3210 (NH), 2932 (C-H aliph.), 1647 (C=O), 1624 (C=O).

^1^H NMR (300 MHz, DMSO-*d_6_*): δ = 2.06 (s, 3H, CH_3_), 2.27 (s, 3H, CH_3_), 3.99, 4.41 (2 s, 2H, CH_2_), 4.53–4.55 (d, 2H, CH_2_, *J* = 6 Hz), 6.52 (s, 1H, H-_pyridine_), 7.47–7.50 (m, 2H, ArH), 7.69–7.72 (m, 2H, ArH), 7.99, 8.17 (2 s, 1H, CH), 8.69 (t, 1H, NH, *J* = 6 Hz), 11.68, 11.78 (2 s, 1H, NH), 13.36 (s, 1H, SH);

^13^C NMR (75 MHz, DMSO-*d_6_*): δ = 18.63, 18.74, 33.33, 33.84, 125.90, 127.67, 129.02, 131.14, 134.31, 136.95, 143.23, 146.93, 148.67, 152.28, 154.42, 161.27, 165.93, 169.62, 173.48, 188.23.

4,6-Dimethyl-*N*-{[4-(2-(2-(4-trifluoromethylbenzylidene)hydrazinyl)-2-oxoethyl)-5-sulfanylidene-4,5-dihydro-1,3,4-oxadiazole-2-yl]methyl}-2-sulfanylpyridine-3-carboxamide **11**.

Yield: 52.2%, m.p.: 253–255 °C.

FT-IR (selected lines, γ_max_, cm^−1^): 3292, 3179 (NH), 2942 (C-H aliph.), 1680 (C=O), 1647 (C=O).

^1^H NMR (300 MHz, DMSO-*d_6_*): δ = 2.06 (s, 3H, CH_3_), 2.27 (s, 3H, CH_3_), 4.02, 4.44 (2 s, 2H, CH_2_), 4.53–4.55 (d, 2H, CH_2_, *J* = 6 Hz), 6.52 (s, 1H, H-_pyridine_), 7.77–7.80 (m, 2H, ArH), 7.88–7.91 (m, 2H, ArH), 8.08, 8.26 (2 s, 1H, CH), 8.71 (t, 1H, NH, *J* = 6 Hz), 11.81, 11.92 (2 s, 1H, NH), 13.38 (s, 1H, SH);

^13^C NMR (75 MHz, DMSO-*d_6_*): δ = 18.54, 18.69, 19.62, 33.45, 33.78, 126.10, 127.72, 128.82, 131.36, 134.45, 136.75, 143.48, 146.76, 148.22, 152.43, 154.65, 161.46, 165.23, 169.42, 173.33, 187.95.

4,6-Dimethyl-*N*-{[4-(2-(2-(3-chlorobenzylidene)hydrazinyl)-2-oxoethyl)-5-sulfanylidene-4,5-dihydro-1,3,4-oxadiazole-2-yl]methyl}-2-sulfanylpyridine-3-carboxamide **12**

Yield: 48.0%, m.p.: 273–275 °C.

FT-IR (selected lines, γ_max_, cm^−1^): 3200 (NH), 2931 (C-H aliph.), 1651 (C=O), 1626 (C=O)

^1^H NMR (300 MHz, DMSO-*d_6_*): δ = 2.06 (s, 3H, CH_3_), 2.27 (s, 3H, CH_3_), 4.00, 4.42 (2 s, 2H, CH_2_), 4.53–4.55 (d, 2H, CH_2_, *J* = 6 Hz), 6.52 (s, 1H, H-_pyridine_), 7.44–7.46 (m, 2H, ArH), 7.64–7.66 (m, 1H, ArH), 7.74–7.76 (m, 1H, ArH), 7.99, 8.17 (2 s, 1H, CH), 8.70 (t, 1H, NH, *J* = 6 Hz), 11.72, 11.85 (2 s, 1H, NH), 13.36 (s, 1H, SH);

^13^C NMR (75 MHz, DMSO-*d_6_*): δ = 18.52, 18.65, 32.13, 33.56, 126.01, 127.23, 128.83, 130.74, 134.62, 137.72, 143.55, 147.12, 148.28, 152.68, 154.89, 161.65, 166.33, 170.42, 172.18, 188.12.

4,6-Dimethyl-*N*-{[4-(2-(2-(2-bromobenzylidene)hydrazinyl)-2-oxoethyl)-5-sulfanylidene-4,5-dihydro-1,3,4-oxadiazole-2-yl]methyl}-2-sulfanylpyridine-3-carboxamide **13**.

Yield: 42.1%, m.p.: 285–288 °C.

FT-IR (selected lines, γ_max_, cm^−1^): 3349, 3205 (NH), 2934 (C-H aliph.), 1664 (C=O), 1648 (C=O).

^1^H NMR (300 MHz, DMSO-*d_6_*): δ = 2.06 (s, 3H, CH_3_), 2.27 (s, 3H, CH_3_), 4.00, 4.43 (2 s, 2H, CH_2_), 4.53–4.55 (d, 2H, CH_2_, *J* = 6 Hz), 6.52 (s, 1H, H-_pyridine_), 7.32–7.34 (m, 1H, ArH), 7.44–7.46 (m, 1H, ArH), 7.66–7.68 (m, 1H, ArH), 7.90–7.92 (m, 1H, ArH), 8.36, 8.54 (2 s, 1H, CH), 8.69 (t, 1H, NH, *J* = 6 Hz), 11.82, 11.99 (2 s, 1H, NH), 13.36 (s, 1H, SH);

^13^C NMR (75 MHz, DMSO-*d_6_*): δ = 18.73, 18.84, 32.59, 34.26, 124.96, 126.58, 129.03, 131.24, 133.67, 137.12, 143.55, 147.65, 147.83, 153.43, 155.68, 161.23, 165.84, 169.62, 171.67, 188.16.

### 3.2. Biological Section

#### 3.2.1. Cell Lines

Six cell lines were used in the investigations. Three of them were normal: NHDF (normal human dermal fibroblasts), purchased from Lonza (Verviers, Belgium), V79 (fibroblasts from Chinese hamster lung) and VERO (kidney epithelial cells) obtained from ECACC (European Collection of Authenticated Cell Cultures). The rest of cells were human cancer cell lines: LoVo (colon adenocarcinoma), their drug resistant subline LoVo/Dx and A549 (pulmonary basial cell alveolar adenocarcinoma) cell line. Cancer lines were also obtained from ECACC.

#### 3.2.2. Cell Culture Conditions

Each cell line was grown in the culture media recommended by supplier. Before the test they were detached with trypsin/EDTA solution, then to neutralize the effect of Trypsin/EDTA solution, FBS containing medium was used. After centrifugation the cells were stained with 0.4% solution of trypan blue, counted and inspected for viability using microscope. In the end, cells were inserted into 96-well culture plates and incubated in CO_2_ incubator (37 °C, 24 h). The number of cells was 2 × 10^3^ cells per well. The tested compounds with different concentrations (5, 10, 20, 50, 100 µM) were dissolved in DMSO and then added to the cells (the final DMSO concentration was 0.1%). The cultures were incubated for another 48 h. After that time the cells were collected to be used in tests.

#### 3.2.3. Cyclooxygenase Inhibitory Activity

A COX Colorimetric Inhibitor Screening Assay Kit, produced by Cayman Chemical Company, Ann Arbor, MI, USA, was used. It used the colorimetric monitoring of oxidized form of TMPD (*N,N,N′,N′*-tetramethyl-*p*-phenylenediamine) which was produced during reduction of prostaglandin G_2_ (PGG_2_) to PGH_2_. The change of colour was observed and measured spectrofotometrically at 590 nm. Reagents used it this assay were: COX-1 and COX-2 enzymes, Tris-HCl buffer, solutions of heme in DMSO and TMPD, arachidonic acid, KOH. To be sure that 100% enzymatic activity was achieved each sample was measured 3 times. The probes were measured after two minutes of incubation with tested compounds in the comparison to the initial activity of enzyme. It allowed to determine IC_50_ value, where 50% inhibition of the enzyme activity was observed.

#### 3.2.4. MTT Assay

MTT assay was used to find out how tested compounds influence the metabolic activity of investigated cell lines. The cells were incubated with tested compounds. After removing of supernatant, 1 mg/mL of MTT solution in MEM was added to the plate (to each well) and then the plates were incubated at 37 °C for 2 h. The medium was removed again, and formazan crystals were dissolved in isopropanol. A Varioscan LUX microplate reader (Thermo Fisher Scientific, Waltham, MA, USA) was used to measure absorbance at 570 nm.

#### 3.2.5. Estimation of Intracellular ROS Level

First, the tested compounds (in concentration of 100 μM) were added to the cell cultures and cells were incubated for 4 h. Then cells were washed and incubated with DCFH-DA (non-fluorescent probe) in dark at 37 °C for 2 h at CO_2_ [79]. Non-fluorescent, non-polar DCFH-DA (2′7′-dichlorodihydrofluorescein diacetate) at concentration of 25 μM was used as a marker of oxidative stress to determine the intracellular ROS levels. Then the cells were washed with PBS two times and 100 μL of H_2_O_2_ were added for 30 min, which is proper time to decompose all H_2_O_2_ [80]. In this time, DCFH-DA which penetrated into the cells, was hydrolyzed by esterases to polar, non-fluorescent DCFH (2′7′-dichlorodihydrofluorescein) and then oxidized in the presence of ROS (reactive oxygen species) to fluorescent DCF (2′7′-dichlorofluorescein). DCF was measured using Varioscan LUX microplate reader (Thermo Fisher Scientific, Waltham, MA, USA) at λ_ex_ = 485 nm and λ_em_ = 535 nm. Then results were presented as E/E_0_ where E is test sample value and E_0_—control value.

#### 3.2.6. Statistics

All results in Tables are presented as mean ± SEM (standard error of the mean) relative to the control (E/E_0_). E is the culture with the tested compound and E_0_ is the negative control without the compound. The routine statistical methods: two-way analysis of variance ANOVA as well as Tukey’s post-hoc test were used. It allowed us to determine the statistical significance of results, where *p* < 0.05 was set as significant. The statistical indicators were estimated using Statistica 13.3 software.

### 3.3. Molecular Modeling—Computational Methodology

Initial models of the investigated compounds (see Figure 1) were constructed in the Molden 6.6 program [81] with the aim of quantum-chemical structural optimization before the main docking runs. The structure of each compound was optimized in accordance with default procedures in the Gaussian 16 suite of programs [82]. Energy minimization was carried out with the ωB97XD functional [83] derived within the framework of density functional theory (DFT) [84,85], with the correlation-consistent Dunning basis set denoted as cc-pVDZ [86]. In order to confirm that the optimized geometries correspond to the energy minima on the potential energy surface (PES), harmonic frequencies calculations were also conducted (yielding no imaginary frequencies). Resulting energy values were used to estimate the conformational preferences. Further, docking runs were carried out for the studied series of compounds. The structures of the receptors were taken from the Protein Data Bank (PDB) [87]. The compounds were docked into COX-1 (PDB code 4O1Z) [76] from *Ovis aries* and COX-2 (PDB code 4M11) [76] from *Mus musculus* models, which were loaded with the ligand (meloxicam, MXM). Additionally, COX-1 (PDB code 6Y3C) [75] and COX-2 (PDB code 5KIR) [77], cyclooxygenases counterparts from *Homo sapiens*, were also taken into consideration as receptors—of these, only the human COX-2 was loaded with rofecoxib (RCX) ligand, while the human COX-1 was in the apo form. Sequence alignments and similarity were generated with the ClustalW software [88]. Water molecules and co-factors that were in proximity to the binding site of receptors were removed. The Gasteiger charges and polar hydrogen atoms were added to the examined receptors and ligands. The flexible parts of the receptors were inferred from the knowledge of the center of co-crystallized ligands and the grid was set to the dimensions of 20 × 20 × 20 Å. The flexible part of the receptor was defined as residues located within 3.5 Å for COX-1 and within 4.0 Å for COX-2 from the centers of the binding pockets. The docking calculations were performed with exhaustiveness of 32 and energy range equal to 10 kcal/mol. The validation of the procedure was carried out with usage of the MXM and RCX ligands from the PDB database and, solely for the purpose to check the validation of the used computational setup, the polar hydrogens were not added. Editing of the protein structure (water and ligand removal) was carried out with the assistance of the VMD 1.9.3 program [89]. The preparation of the ligand and receptor, molecular docking and the visualization of the results were performed with the AutoDockTools 1.5.7 [90], AutoDock Vina 1.1.2 [91] and the open-source PyMOL 2.3.0 [92] packages. The 2D diagrams were prepared in the Discovery Studio Visualizer 2019 [78] with the use of the most energetically favorable geometries of the examined compounds, and the script vina_split of the AutoDock Vina 1.1.2 program [91] was used to split merged conformations from one PDBQT file into separate entries.

## 4. Conclusions

In the current study, we have presented experimental and theoretical results obtained for new Schiff base-type compounds. The reaction pathways and synthetic procedures have been presented. The physico-chemical properties of the compounds were characterized by NMR and IR spectroscopy. The biological activity was investigated based on diverse biological assays. It was found that Schiff base **13** inhibited the activity of both isoenzymes, COX-1 and COX-2 at a lower concentration than standard drugs, and its COX-2/COX-1 selectivity ratio was detected to be similar to meloxicam. The results of cytotoxicity assay showed that all of the tested compounds exhibited potent anti-cancer activity against A549, MCF-7, LoVo, and LoVo/Dx cell lines.

Finally, the quantum-chemical based DFT method simulations were performed. Two main possible conformations were investigated, and it was found that lower relative energy corresponded to the sandwich-like structure. As the last step of the study, the flexible docking method was applied and it was found that in comparison to known inhibitors of cyclooxygenases and affinity energies of docking meloxicam, piroxicam and rofecoxib to the receptors were similar to most of the energies obtained for the investigated structures. These results indicate that the examined ligands could exhibit a significant activity towards human COX-1 and COX-2 receptors.

Considering the results obtained, we wish to underline that the combination of 1,3,4-oxadiazole and hydrazide-hydrazone pharmacophore moieties has yielded promising results, and additional studies on these compounds are necessary. The research into their effects on apoptosis, the cell cycle and inflammation is planned to improve our understanding of their mechanisms of action.

## Figures and Tables

**Table 1:** IC_50_ values and mean SD for COX-1 and COX-2 activities after 2 min of incubation with tested compounds, *n* = 5.

Compound	IC_50_ [µM] ± SD		COX-2/COX-1 Selectivity Ratio
	COX-1	COX-2	
**4**	135.20 ± 5.99	NA	-
**5**	73.91 ± 3.03	186.95 ± 15.25	2.53
**6**	144.79 ± 20.88	173.93 ± 5.84	1.20
**7**	147.55 ± 19.81	184.43 ± 18.84	1.25
**8**	156.65 ± 12.12	NA	-
**9**	153.89 ± 6.29	102.58 ± 10.61	0.67
**10**	NA	127.54 ± 1.87	-
**11**	86.19 ± 2.60	169.75 ± 19.14	1.97
**12**	66.84 ± 5.19	90.71 ± 19.61	1.36
**13**	84.37 ± 5.89	73.23 ± 5.66	0.80
Piroxicam	87.44 ± 4.17	80.11 ± 1.26	0.92
Meloxicam	128.76 ± 4.72	76.38 ± 6.46	0.59

Note: Data are shown as a mean ± standard deviation, NA stands for “not applicable”.

**Table 2:** MTT results—NHDF, VERO, V79, A459, MCF-7, LoVo and LoVo/Dx IC_50_ (µM) values and mean SD for the studied compounds.

Compound	IC_50_ [µM] ± SD						
	NHDF	VERO	V79	A549	MCF-7	LoVo	LoVo/Dx
**4**	57.46 ± 1.22	77.44 ± 5.84	71.70 ± 5.80	3.84 ± 0.30	9.63 ± 1.66	3.01 ± 0.20	5.73 ± 3.84
**5**	58.97 ± 1.49	100.1 ± 13.49	84.30 ± 3.40	3.62 ± 0.43	6.37 ± 1.09	3.13 ± 0.17	5.69 ± 1.03
**6**	58.21 ± 0.92	59.62 ± 5.42	80.70 ± 5.60	4.05 ± 0.26	6.37 ± 0.19	3.20 ± 0.26	7.07 ± 2.48
**7**	58.23 ± 2.00	68.31 ± 3.24	47.70 ± 1.73	3.72 ± 0.54	6.33 ± 0.56	3.46 ± 0.39	7.89 ± 1.78
**8**	58.69 ± 1.94	50.38 ± 2.45	71.10 ± 2.40	4.34 ± 0.56	9.41 ± 4.80	3.60 ± 0.55	21.00 ± 5.92
**9**	59.44 ± 1.90	89.76 ± 8.43	77.70 ± 4.41	3.36 ± 0.20	8.22 ± 5.98	3.24 ± 0.10	6.87 ± 1.45
**10**	59.90 ± 2.04	129.8 ± 9.50	56.10 ± 5.42	3.52 ± 0.19	6.19 ± 0.19	3.12 ± 0.23	22.31 ± 2.10
**11**	58.99 ± 3.62	104.5 ± 4.98	55.20 ± 2.75	3.72 ± 0.24	8.29 ± 4.45	3.12 ± 0.31	17.50 ± 8.73
**12**	58.73 ± 2.21	79.2 ± 14.44	52.80 ± 7.20	3.88 ± 0.50	9.04 ± 2.84	3.07 ± 0.21	22.56 ± 8.19
**13**	59.26 ± 3.43	55.66 ± 1.84	65.40 ± 4.04	3.16 ± 0.21	7.23 ± 0.49	2.89 ± 0.08	9.40 ± 5.97
Piroxicam	162.23 ± 22.85	NA	213.10 ± 23.25	110.12 ± 28.23	NA	122.16 ± 10.20	125.50 ± 11.23
Meloxicam	195.66 ± 35.22	NA	200.80 ± 17.80	148.30 ± 27.58	NA	129.56 ± 8.80	142.30 ± 9.46

Note: Data are shown as a mean ± standard deviation, NA stands for “not applicable”.

**Table 3:** Therapeutic index calculated by ratio of concentrations that inhibit 50% of healthy and cancerous cells.

Compound	Therapeutic Index			
	NHDF/A549	NHDF/MCF-7	NHDF/LoVo	NHDF/LoVo/Dx
**4**	14.40 ± 0.70 ^a^	6.16 ± 0.98 ^a^	18.26 ± 1.20 ^a^	8.97 ± 3.21
**5**	15.82 ± 1.78 ^a^	9.29 ± 1.29 ^a^	16.93 ± 1.34 ^a^	7.60 ± 1.50 ^a^
**6**	13.64 ± 1.32 ^a^	9.21 ± 0.03 ^a^	16.51 ± 2.01 ^a^	2.94 ± 0.77 ^a^
**7**	17.71 ± 0.49 ^a^	9.42 ± 0.54 ^a^	18.35 ± 0.02 ^a^	8.88 ± 1.63 ^a^
**8**	17.03 ± 0.34 ^a^	7.76 ± 4.27	19.24 ± 0.77 ^a^	2.70 ± 0.16 ^a^
**9**	15.86 ± 0.05 ^a^	12.10 ± 5.01	18.96 ± 0.73 ^a^	4.02 ± 2.04
**10**	15.26 ± 1.41 ^a^	9.49 ± 0.07 ^a^	19.16 ± 0.59 ^a^	2.84 ± 1.00
**11**	18.76 ± 0.16 ^a^	8.87 ± 5.03	20.49 ± 0.62 ^a^	8.89 ± 3.50
**12**	1.50 ± 0.18 ^a^	18.67 ± 3.51 ^a^	1.32 ± 0.07 ^a^	1.29 ± 0.07 ^a^
**13**	1.32 ± 0.01 ^a^	26.92 ± 3.05 ^a^	1.50 ± 0.17 ^a^	1.37 ± 0.16
Piroxicam	15.01 ± 0.86 ^a^	-	19.13 ± 0.87 ^a^	15.31 ± 6.67
Meloxicam	16.41 ± 1.55 ^a^	-	18.86 ± 0.55 ^a^	10.57 ± 1.68 ^a^

Note: Data are shown as a mean ± standard deviation. ^a^ Represents the significance level at *p* < 0.05.

**Table 4:** ROS scavenging activity of tested compounds on V79 cell line. The results were expressed as E/E_0_ ratios, where E_0_ are the control samples without tested compounds, *n* = 5.

Compound	without H_2_O_2_	with H_2_O_2_
	Mean E/E_0_ ± SD	
**4**	0.53 ± 0.22 ^a^	0.42 ± 0.08 ^a^
**5**	0.82 ± 0.13	0.59 ± 0.21 ^a^
**6**	0.82 ± 0.17	0.97 ± 0.11
**7**	1.03 ± 0.39	0.81 ± 0.23
**8**	0.85 ± 0.29	0.72 ± 0.11 ^a^
**9**	0.78 ± 0.31	0.71 ± 0.12 ^a^
**10**	0.73 ± 0.27	0.82 ± 0.37
**11**	0.67 ± 0.24	1.12 ± 0.20
**12**	0.68 ± 0.27	0.62 ± 0.25
**13**	0.59 ± 0.08 ^a^	0.50 ± 0.15 ^a^
Piroxicam	0.98 ± 0.17	0.67 ± 0.17 ^a^
Meloxicam	1.15 ± 0.19	0.85 ± 0.18
Ascorbic acid	0.83 ± 0.13	0.22 ± 0.12 ^a^
Trolox	0.71 ± 0.05 ^a^	0.15 ± 0.08 ^a^

Note: Data are shown as a mean ± standard deviation. ^a^ Represents the significance level at *p* < 0.05.

**Table 5:** ROS scavenging activity of tested compounds on LoVo cell line. The results were expressed as E/E_0_ ratios, where E_0_ are the control samples without tested compounds, *n* = 5.

Compound	without H_2_O_2_	with H_2_O_2_
	Mean E/E_0_ ± SD	
**4**	0.69 ± 0.33	0.83 ± 0.08 ^a^
**5**	0.72 ± 0.21	0.87 ± 0.21
**6**	0.74 ± 0.22	0.88 ± 0.04 ^a^
**7**	1.07 ± 0.38	0.95 ± 0.27
**8**	0.65 ± 0.20 ^a^	0.80 ± 0.22
**9**	0.76 ± 0.22	0.91 ± 0.19
**10**	0.85 ± 0.27	0.91 ± 0.14
**11**	1.24 ± 0.65	0.84 ± 0.17
**12**	0.91 ± 0.43	0.79 ± 0.16
**13**	0.70 ± 0.30	0.69 ± 0.10^a^
Piroxicam	0.72 ± 0.19	0.81 ± 0.19
Meloxicam	1.06 ± 0.32	0.89 ± 0.16
Ascorbic acid	0.89 ± 0.11	0.32 ± 0.09 ^a^
Trolox	0.85 ± 0.13	0.29 ± 0.15 ^a^

Note: Data are shown as a mean ± standard deviation. ^a^ Represents the significance level at *p* < 0.05.

**Table 6:** The binding affinity energy obtained for the studied compounds interacting with the COX-1 (PDB code 6Y3C) receptor.

COX-1 [75]			
Compound	Affinity Energy [kcal/mol]	Compound	Affinity Energy [kcal/mol]
**4(E)**	−6.7	**4(S)**	−6.0
**5(E)**	−7.5	**5(S)**	−8.2
**6(E)**	−7.0	**6(S)**	−8.0
**7(E)**	−7.0	**7(S)**	−8.2
**8(E)**	−7.6	**8(S)**	−7.8
**9(E)**	−6.8	**9(S)**	−8.3
**10(E)**	−7.8	**10(S)**	−7.9
**11(E)**	−8.7	**11(S)**	−8.5
**12(E)**	−7.0	**12(S)**	−8.2
**13(E)**	−8.3	**13(S)**	−8.3
RCX/MXM	−8.1/−6.4	Piroxicam	−7.5

**Table 7:** The binding affinity energy obtained for the studied compounds interacting with the COX-1 (PDB code 4O1Z) receptor.

COX-1 [76]			
Compound	Affinity Energy [kcal/mol]	Compound	Affinity Energy [kcal/mol]
**4(E)**	−8.4	**4(S)**	−8.0
**5(E)**	−10.1	**5(S)**	−10.2
**6(E)**	−10.2	**6(S)**	−10.4
**7(E)**	−10.7	**7(S)**	−10.6
**8(E)**	−9.8	**8(S)**	−9.5
**9(E)**	−10.4	**9(S)**	−10.7
**10(E)**	−8.6	**10(S)**	−9.5
**11(E)**	−9.8	**11(S)**	−10.3
**12(E)**	−7.9	**12(S)**	−8.3
**13(E)**	−8.7	**13(S)**	−8.3
RCX/MXM	−9.5/−9.1	Piroxicam	−9.7

**Table 8:** The binding affinity energy obtained for the studied compounds interacting with the COX-2 (PDB code 4M11) receptor.

COX-2 [76]			
Compound	Affinity Energy [kcal/mol]	Compound	Affinity Energy [kcal/mol]
**4(E)**	−8.8	**4(S)**	−9.0
**5(E)**	−10.0	**5(S)**	−10.1
**6(E)**	−10.6	**6(S)**	−10.4
**7(E)**	−10.7	**7(S)**	−10.6
**8(E)**	−10.1	**8(S)**	−10.1
**9(E)**	−10.6	**9(S)**	−10.9
**10(E)**	−10.3	**10(S)**	−10.8
**11(E)**	−11.5	**11(S)**	−10.8
**12(E)**	−10.2	**12(S)**	−10.6
**13(E)**	−10.4	**13(S)**	−10.6
RCX/MXM	−10.3/−10.0	Piroxicam	−10.4

**Table 9:** The binding affinity energy obtained for the studied compounds interacting with the COX-2 (PDB code 5KIR) receptor.

COX-2 [77]			
Compound	Affinity Energy [kcal/mol]	Compound	Affinity Energy [kcal/mol]
**4(E)**	−9.3	**4(S)**	−9.4
**5(E)**	−10.8	**5(S)**	−9.4
**6(E)**	−9.8	**6(S)**	−10.2
**7(E)**	−10.0	**7(S)**	−9.5
**8(E)**	−9.6	**8(S)**	−9.1
**9(E)**	−10.1	**9(S)**	−9.6
**10(E)**	−10.0	**10(S)**	−9.4
**11(E)**	−11.4	**11(S)**	−10.2
**12(E)**	−10.2	**12(S)**	−9.7
**13(E)**	−10.8	**13(S)**	−10.9
RCX/MXM	−10.9/−9.9	Piroxicam	−9.8

## Supplementary Materials

The following are available online at https://www.mdpi.com/article/10.3390/ijms23010549/s1.
