# Supplementary material for: Biological Evaluation and Molecular Docking Studies of Novel 1,3,4-Oxadiazole Derivatives of 4,6-Dimethyl-2-sulfanylpyridine-3-carboxamide
Source: Int J Mol Sci. 2022 Jan 4;23(1):549. doi: 10.3390/ijms23010549 (PMC8745710; doi:10.3390/ijms23010549)
Supplement: Supplementary file 1 [file ijms-23-00549-s001.zip › ijms-1513376-supplementary.pdf]

Supplementary materials

Biological Evaluation and Molecular Docking Studies of Novel 1,3,4-Oxadiazole Derivatives of 4,6-Dimethyl-2-Sulfanylpyridine-3-Carboxamide

Piotr Świątek [1*], Teresa Glomb [1*], Agnieszka Dobosz [2], Tomasz Gębarowski [3], Kamil Wojtkowiak [4], Aneta Jezierska [4], Jarosław J. Panek [4], Małgorzata Świątek [5], Małgorzata Strzelecka [1]

[1] Department of Medicinal Chemistry, Faculty of Pharmacy, Wroclaw Medical University, Borowska 211, 50-556 Wroclaw, Poland; malgorzata.strzelecka@umw.edu.pl (M.S.)

[2] Department of Medical Science Foundation, Faculty of Pharmacy, Wroclaw Medical University, Borowska 211, 50-556 Wroclaw, Poland; agnieszka.dobosz@umw.edu.pl (A.D.)

[3] Department of Biostructure and Animal Physiology, Wroclaw University of Environmental and Life Sciences, Kożuchowska 1/3, 51-631 Wroclaw, Poland; tomasz.gebarowski@upwr.edu.pl (T.G.)

[4] University of Wroclaw, Faculty of Chemistry, F. Joliot-Curie 14, 50-383 Wrocław, Poland; jaroslaw.panek@chem.uni.wroc.pl (J.J.P.); aneta.jezierska@chem.uni.wroc.pl (A.J.); kamil.wojtkowiak@chem.uni.wroc.pl (K.W.)

[5] Hospital Pharmacy, University Clinical Hospital, Borowska 213, 50-556 Wrocław, Poland; mswiatek@usk.wroc.pl (M.Ś)

* Correspondence: piotr.swiatek@umw.edu.pl (P.Ś.); teresa.glomb@umw.edu.pl (T.G.), Tel.: +48-717840391

(a)

CLUSTAL 2.1 MULTIPLE SEQUENCE ALIGNMENT

Ovine COX-1 (4O1Z) vs. human COX-1 (6Y3C): 91% sequence similarity

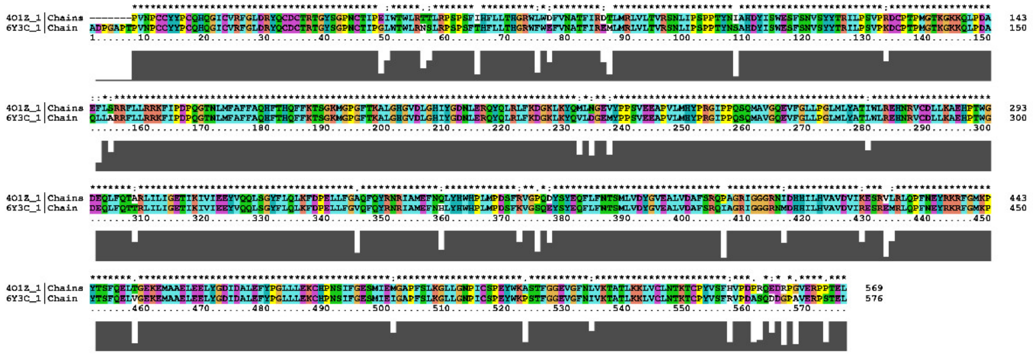

(b)

CLUSTAL 2.1 MULTIPLE SEQUENCE ALIGNMENT

Murine COX-2 (4M11) vs. human COX-2 (5KIR): 88% sequence similarity

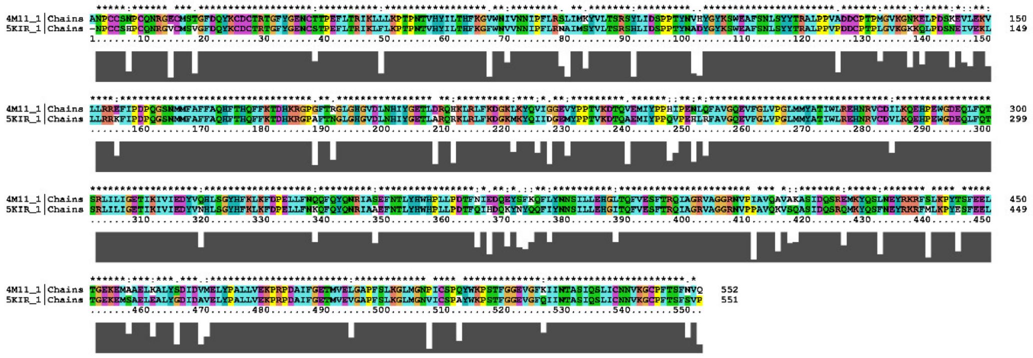

(c)

CLUSTAL 2.1 MULTIPLE SEQUENCE ALIGNMENT

Human COX-1 (6Y3C) vs. human COX-2 (5KIR): 66% sequence similarity

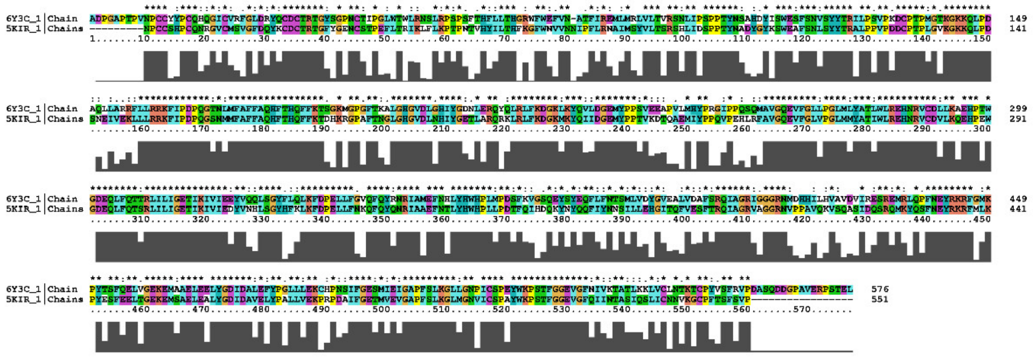

Figure S1. Sequence alignment of the proteins used in the study: (**a**) ovine COX-1 vs. human COX-1, (**b**) murine COX-2 vs. human COX-2, and (**c**) human COX-1 vs. human COX-2.

Table S1. The SCF energies for two series of examined compounds calculated at the ωB97XD/cc-pVDZ level of theory.

**Total and relative energy of the structures**				
**Compound**	**SCF energy [kcal/mol]**	**Compound**	**SCF energy [kcal/mol]**	**Relative energy of E vs. S [kcal/mol]**
**4(E)**	-1164210.45	**4(S)**	-1164209.94	-0.5
**5(E)**	-1333053.63	**5(S)**	-1333063.04	9.4
**6(E)**	-1395310.96	**6(S)**	-1395321.67	10.7
**7(E)**	-1357719.42	**7(S)**	-1357729.16	9.7
**8(E)**	-1607580.85	**8(S)**	-1607590.47	9.6
**9(E)**	-1390917.34	**9(S)**	-1390928.55	11.2
**10(E)**	-1621455.36	**10(S)**	-1621464.66	9.3
**11(E)**	-1544506.58	**11(S)**	-1544516.83	10.3
**12(E)**	-1621455.04	**12(S)**	-1621464.31	9.3
**13(E)**	-2947974.15	**13(S)**	-2947984.65	10.5

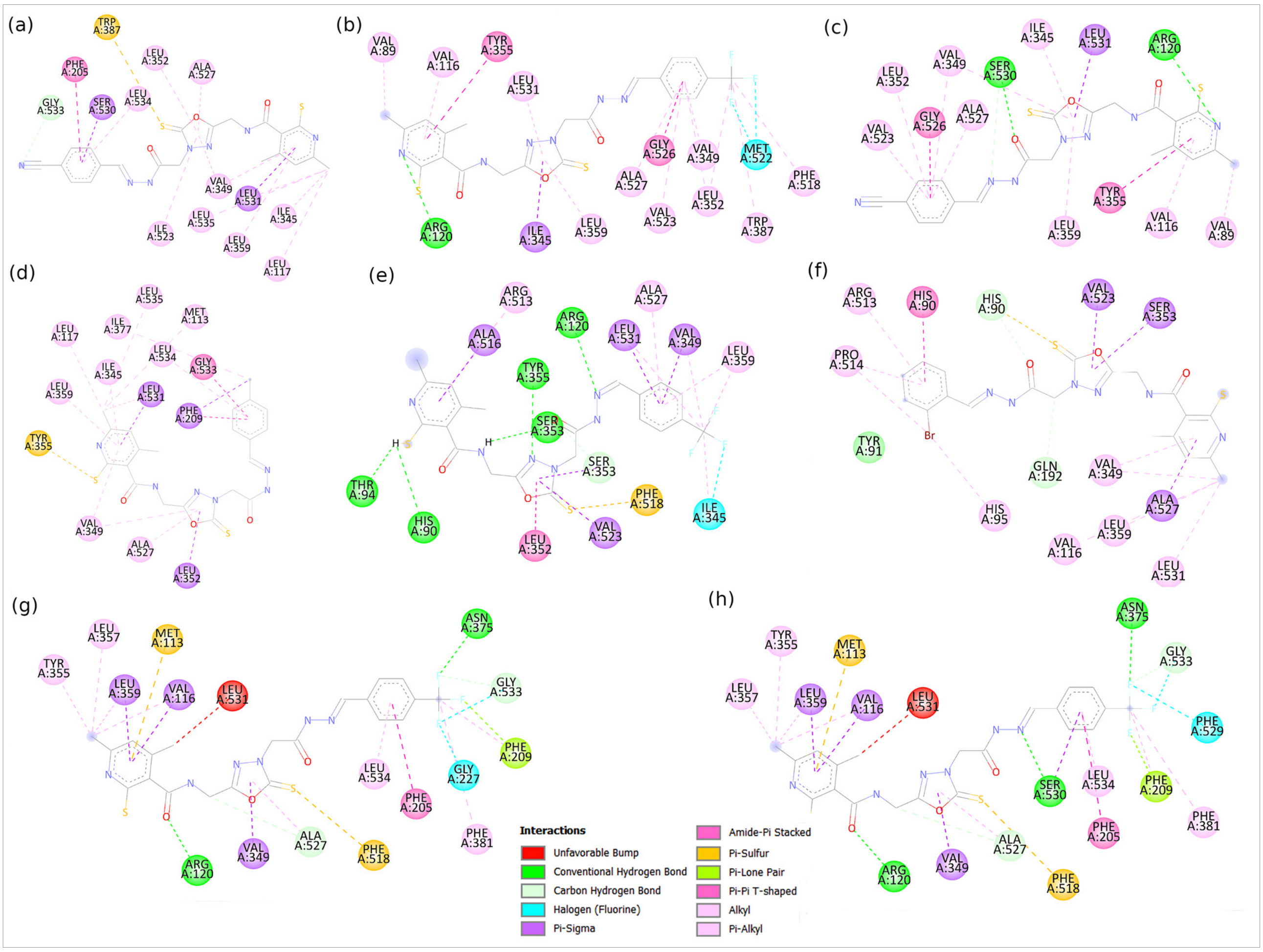

Figure S2. Non-covalent interactions 2D diagram for studied compounds in receptor binding sites: (**a**) **9(S)** in ovine COX1, (**b**) **11(E)** in murine COX2, (**c**) **9(S)** in murine COX2, (**d**) **7(E)** in ovine COX1, (**e**) **11(E)** in human COX2, (**f**) **13(S)** in human COX2, (**g**) **11(S)** in human COX1, (**h**) **11(E)** in human COX1.
